# Reviewed article: Muñoz, FL; Cruz, RF; Silveira, LVA, Diaz, MP. Latent dimension of comorbidities and depression in older adults: a cross-sectional study using National Health Survey data, Brazil, 2019. Epidemol Serv Saude. 2026:35;e20250722

**DOI:** 10.1590/S2237-96222026v35e20250722.a

**Published:** 2026-10-12

**Authors:** Fabiana Maria de Aguiar Bello Marques

**Affiliations:** 1 Secretaria de Saúde do Recife, Recife, PE, Brazil Secretaria de Saúde do Recife Recife PE Brazil

## Peer review A

### Questionnaire

Does the manuscript contain new and significant information to justify publication? Yes 

Does the Abstract (Summary) clearly and accurately describe the content of the article? Yes 

Is the problem significant and concisely stated? Yes 

Are the methods described comprehensively? Yes 

Are the interpretations and conclusions justified by the results? Yes 

Is adequate reference made to other work in the field? Yes 

### Manuscript Structure:

Length of article is: Adequate

Number of tables is: Adequate

Number of figures is: Adequate

### Rating

Interest

Quality: Good

Originality: Good

Overall: Good

Recommendation: Minor Revision 

### Comments

Prezados (as) autores(as),

Agradeço a oportunidade de revisar este manuscrito, que aborda um tema relevante e atual. A seguir, apresento observações destinadas a aprimorar a qualidade e a clareza do trabalho.

1. Objetivos e hipóteses

O objetivo está bem delimitado, mas recomenda-se explicitar de forma mais clara a hipótese central a ser testada com o uso do modelo proposto.

2. Delineamento e metodologia

O delineamento transversal foi bem justificado. A descrição da amostragem e das variáveis está completa.

3. Análise estatística

O uso do modelo de equações estruturais generalizadas é pertinente ao objetivo e agrega valor científico. A razão de prevalência foi corretamente usada em detrimento da odds ratio, o que é adequado para estudos transversais.

4. Resultados e tabelas

As tabelas estão bem organizadas, mas na Tabela 1 (p. 14 do PDF) recomenda-se ajustar os termos “Si” para “Sim” nos itens DAC e AVC.

Figura 3 (modelo para mulheres) está visualmente clara, mas sugere-se incluir legenda mais explicativa, voltada ao leitor não especialista.

5. Discussão e conclusão

A discussão está bem fundamentada, com referências atualizadas e coerência com os resultados.

Seria interessante ampliar a discussão sobre a aplicabilidade dos achados para formulação de estratégias de cuidado no SUS, considerando o envelhecimento populacional.

6. Coerência textual e linguagem

O texto apresenta boa fluidez e precisão terminológica.

Sugestão de revisão leve quanto à redundância de algumas expressões e uso excessivo de conjunções explicativas (ex: “além disso”).

7. Referências

As referências estão majoritariamente de acordo com as normas Vancouver.

No entanto, recomenda-se:

Atualizar referências, quando possível, para incluir trabalhos de 2023 e 2024 relacionados ao tema (há publicações recentes sobre comorbidade e depressão em idosos no contexto pós-COVID-19).

Recomendações finais

A recomendação é revisão menor (minor revision), com ajustes formais e clarificações metodológicas pontuais, além da revisão de detalhes na tabela e nas referências.

